# Assessing the competitiveness of medical humanities research on psychiatry, otolaryngology, and ophthalmology residency program applications

**DOI:** 10.1080/10872981.2023.2212929

**Published:** 2023-05-11

**Authors:** Jasmine Leahy, Jason Joonho Jo, William Steidl, Jacob Appel

**Affiliations:** a Icahn School of Medicine at Mount Sinai, New York, NY, USA; b Department of Psychiatry, Mount Sinai Hospital, New York, NY, USA

**Keywords:** Medical humanities, residency, medical education, psychiatry, ophthalmology, otolaryngology, postgraduate medical education

## Abstract

Medical humanities research is an increasing area of interest for students as medical schools become more aware of the benefits of humanities and the arts on patient care. However, medical students may feel dissuaded from pursuing medical humanities work for fear of how it will be perceived on their residency applications. In this study, residency program directors (PDs) in New York state in psychiatry, ophthalmology, and otolaryngology were surveyed about their opinions on the competitiveness of students doing medical humanities research applying to their programs. Of the 64 PDs contacted, twenty submitted responses (31.3%). When asked if a residency applicant who only had medical humanities research experience would be seriously considered for their program, 95% of PDs said yes. Furthermore, 65% of PDs said that having medical humanities research experience in addition to clinical research increased a student’s chance of being accepted to their program. Thirty percent of PDs indicated that the medical humanities were an important selection criteria for their program. Qualitative responses emphasized that non-traditional projects, such as personal essays, were as valid as published journal articles when conducted with academic rigor. Many PDs also believed that the medical humanities increased compassion, empathy, and communication skills in their residents. Considering these results, medical students should feel empowered to pursue medical humanities research, even if they are applying into a competitive surgical specialty. It should not diminish their chances of being seriously considered for a program, and may even confer an advantage over their clinical research peers.

## Introduction

Research in the medical humanities investigates the connection between medicine and humanities fields like philosophy, history, literature, anthropology, law, music, and art. The scholarly pursuit of medical humanities can take many forms beyond the standard scientific paper, including essays, poems, visual art, music compositions, and workshops.

In general, medical humanities as an academic discipline has become an increasingly important part of medical school education [1–3]. In many cases, it is now a required component of the curriculum [1]. Some have even argued that undergraduate humanities majors are the ideal candidates for medical school admissions because they may possess a more compassionate and nuanced understanding of the human condition [3]. Early medical school admission programs, such as the Flexmed program at the Icahn School of Medicine at Mount Sinai, relieve accepted undergraduate students of premedical requirements so they may pursue humanities interests, reflecting a shift towards person-centered care and a holistic application review process. Along the same line, the Medical College Admission Test (MCAT) as of 2015 has put a stronger emphasis on humanities and social sciences [4]. Although residency programs have begun incorporating elements of narrative medicine and storytelling into their education, it remains to be seen whether the residency admissions process embraces medical humanities on the same level as medical schools and undergraduate institutions [5].

As residency placement becomes more competitive each year and traditional metrics, such as the United States Medical Licensing Exam (USMLE) Step 1 and pre-clinical course performance, switch to pass/fail grading systems, medical students are concerned with what residency programs want to see in their applicants. Previous studies have attempted to tease out the importance of research on residency applications with varying results. While research consistently ranks among one of the most important factors on an application [6,7], it has been shown that research quantity beyond one first author publication is not a significant factor in matching [8]. However, the high number of students who misrepresent their research output combined with the large increase in medical student research activity in the last decade suggests that students consider it a top priority [8,9].

One of the key questions students are faced with in medical school is what type of research they want to pursue. The cultural shift away from objective scores and metrics towards holistic application review raises important questions about the perception of non-clinical research, including the medical humanities. While the medical humanities are solidly embraced on the university level and increasingly so on the medical school level, residency programs have been slow to adopt them [2,10,11]. Furthermore, researchers are still searching for a truly effective, quantitative method of measuring the impact of medical humanities on students [12,13]. As a result, students may express concern that pursuing medical humanities activities detracts from their competitiveness in residency, especially if it is at the cost of not pursuing clinical research.

To our knowledge, no prior study exists that directly asks residency program directors (PDs) for their perception of medical humanities research on program applications. We used a mixed methods approach to both satisfy the current lack of quantitative data existing on medical humanities and to capture the nuances that the humanities requires. We hypothesize that doing medical humanities research will not preclude students from being seriously considered for residency programs in either surgical and medicine fields, and may even help students stand out among their peers. It is our hope that this study alleviates hesitations that students may feel about pursuing medical humanities research during medical school.

## Materials and methods

Our study surveyed residency PDs across New York State in both surgical and medicine fields (otolaryngology, ophthalmology, and psychiatry) about their opinion on the relative competitiveness of residency program applicants who completed medical humanities research as opposed to clinical research. Additionally, we probed what directors perceived to be the benefits, if any, of medical humanities in being a better doctor in residency and beyond. Ophthalmology and otolaryngology were selected because of their perceived competitiveness and limited residency spots. Psychiatry was chosen as a contrast to these two fields as it is known to attract more open-minded and humanities-inclined applicants [14,15].

We completed this descriptive study via administration of a 5-question online survey on Google Forms (Google LLC) to residency PDs of the 2022–2023 academic year. All PDs of New York State residency programs were identified using residency program websites. A total of 64 PDs were emailed with an invitation to the survey, with two subsequent follow-up reminder emails. There was a total of 37 psychiatry PDs, 16 ophthalmology PDs, and 11 otolaryngology PDs. Compensation was not provided, and participation was voluntary. Neither patients nor the public were involved in the design, conduct, reporting, or dissemination plans of our research.

The surveys were composed of four multiple choice response questions and one open-ended response question. An optional question was left for additional comments. Questions were designed to optimize ease of completion for the residency PDs and utility for potential residency program applicants in determining the positive, neutral, or negative effect of medical humanities research on residency application competitiveness. Three types of questions, dichotomous, Likert scale, and open-ended, were incorporated to maximize efficiency as well as leave space for optional expression. No names or demographic variables were recorded. All questions are included in the Results section verbatim. All questions, both quantitative and qualitative, were recorded and analyzed based on number and sentiment by the authors using Google Sheets (Google LLC). For the open-ended questions, the authors categorized the responses based on perceived sentiment (positive, neutral, or negative) on the effect of humanities research on residency applicant competitiveness.

## Results

A total of 20 PDs completed the survey (31.3%). Of these, ten (27.0%) were from psychiatry, six (37.5%) from ophthalmology, and four (36.3%) from otolaryngology. The responses to the first four questions are graphically represented in Figure 1a,b.

**Figure 1.**
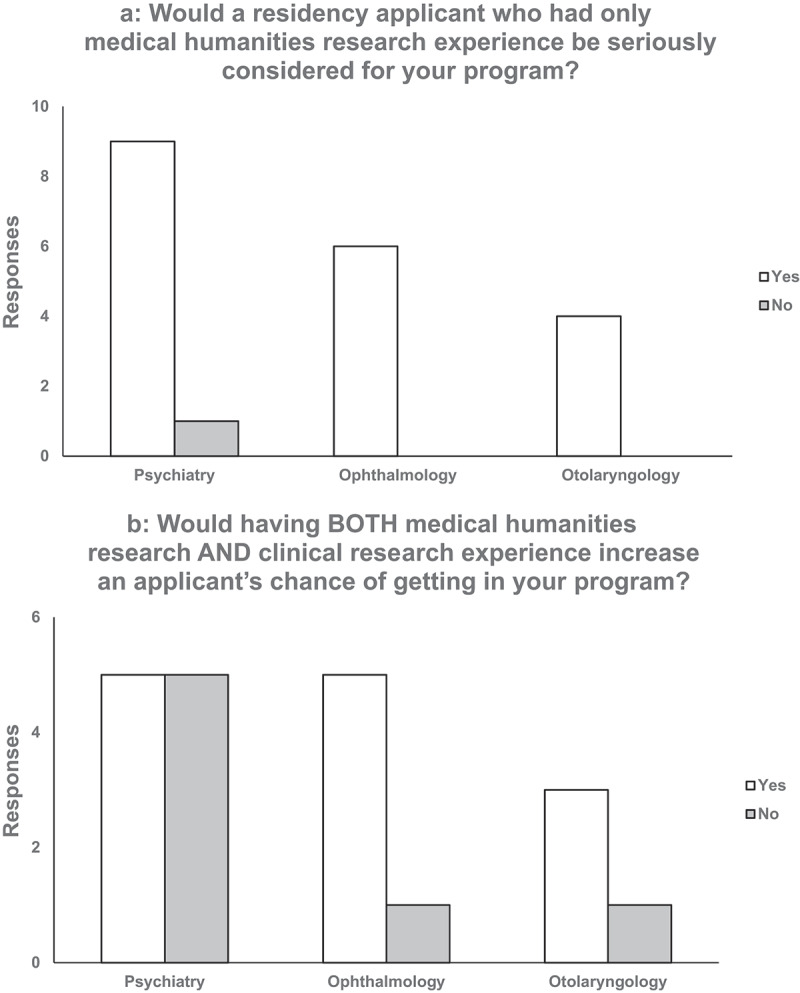
(a) Question 1 on survey. (b) Question 2 on survey (C) Question 3 on survey. (d) Question 4 on survey.

The fifth question, ‘How do you think medical humanities research and/or coursework affects an individual’s performance in residency and beyond?’ yielded a variety of responses. All are included in Table 1 based on perceived positive, neutral, or negative sentiment on the effect of humanities research on residency applications. Notable positive responses included ‘Possibly that resident is more compassionate or takes in effect the psychosocial impact on healthcare’ and ‘Yes I think it can inform a better understanding of the humanistic aspects of being a doctor and psychiatrist.’ Notable neutral responses included ‘No effect’ and ‘Very little. It may make the individual more interesting, but unlikely to improve their mastery of diagnosis and treatment.’ Significantly, no responses were identified to express that medical humanities research would negatively affect a residency applicant’s competitiveness.

**Table 1.** Responses to question 5, How do you think medical humanities research and/or coursework affects an individual’s performance in residency and beyond.

Sentiment	Psychiatry	Ophthalmology	Otolaryngology
Positive	‘Yes I think it can inform a better understanding of the humanistic aspects of being a doctor and psychiatrist’ ‘Increases empathy, possibly psychological mindedness’ ‘Improves quality of care’ ‘Any broader perspective for understanding the human experience can be helpful in one’s development as a physician.’ ‘A lot. It broadens the view of the individual’ ‘It affects applicants analytic skills and improves psychological mindedness.’ ‘It is a good indication of an individual’s interest in humanism, which is an important part of being an effective clinician.’	‘I think it is a nice addition to their other coursework, but do not think it is as important as their basic science coursework or research.’ ‘It’s probably situation-dependent [sic] but I would imagine any sustained thought on that subject would be beneficial for the individual and resident group as a whole.’ ‘Ability to understand patients and (hopefully) be more patient with colleagues/staff/stakeholders in a teaching/service environment’ ‘I like to see a thoughtful applicant’	‘Possibly that resident is more compassionate or takes in effect the psychosocial impact on healthcare’ ‘It most often helps with patient dialogue and understanding of diversity’ ‘Broadens views, matures applicant, allows to connect with different patient populations; ideally!’
Neutral	‘It depends on what the focus is.’ ‘No impact on residency application but may well be helpful caring for patients’ ‘Very little. it may make the individual more interesting, but unlikely to improve their mastery of diagnosis and treatment.’	‘Unknown’	‘No effect’
Negative			

Question 6 offered PDs a space to expound upon question 5. A total of six PDs (6/20, 30%) filled out this optional question. Of these three (3/10, 30%) psychiatry, two (2/6, 33%) ophthalmology, and one (1/4, 25%) otolaryngology PD submitted responses. One psychiatry PD noted that ‘one type of research does not weigh more strongly than another.’ One ophthalmology PD echoed this sentiment, writing that ‘on some level, research is research.’ Moreover, they added that they ‘especially enjoy talking to the students about their research in these domains.’ Conversely, a second ophthalmology PD wrote that while humanities research is a ‘nice addition,’ it was not as important as ‘basic science coursework or research.’ All responses are presented in Table 2 separated based on positive, neutral, or negative sentiment.

**Table 2.** Optional responses to question 6, [Optional] Any further elaboration on how medical humanities research affects the relative competitiveness of a residency application in your specialty.

Sentiment	Psychiatry	Ophthalmology	Otolaryngology
Positive	‘We do a holistic review of applications so any research experience is strongly considered and one type of research does not weigh more strongly than another. We feel commitment to a project and completion is more important than quantity.’ ‘Doing work related to humanities/creativity/writing/art does not have to involve “research” on medical humanities for it to be a valuable experience that we look for in applicants to residency. In other words, we would value the work of someone who published a personal essay as much as someone who did medical humanities research.’	‘On some level, research is research. It implies that a student devoted a certain amount of time to the betterment of our profession (and themselves). They took a project from concept to publication. That’s a challenge and a feat. Thus I would see it in the same way I view clinical research. That said, I especially enjoy talking to the students about their research in these domains.’ ‘I think it is a nice addition to their other coursework, but do not think it is as important as their basic science coursework or research.’	
Neutral	‘Our program looks for individuals who are likely to pass the ABPN boards, and who will fit in well with our residents and staff.’		‘Does not effect [sic] relative competitiveness’
Negative			

## Discussion

Our results support that applicants with only medical humanities research background may be seriously considered for psychiatry, ophthalmology, and otolaryngology residency programs (Figure 1a). We conclude that students participating in medical humanities research should not be concerned about their relative competitiveness compared to their clinical research peers. In fact, the survey suggests that medical humanities research in addition to clinical research may actually give an edge to applicants (Figure 1a).

**Figure 1.**
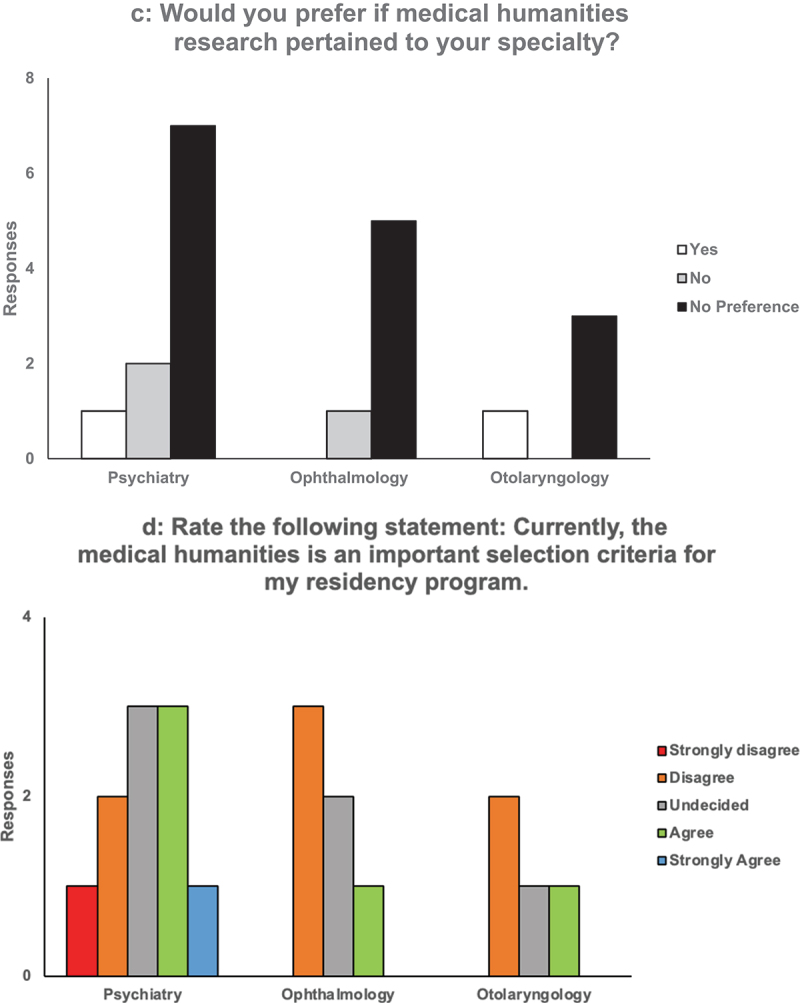
(Continued).

Many residency PDs expressed value in the medical humanities, saying that they encouraged more open-mindedness and empathy (‘resident is more compassionate,’ ‘increases empathy’; Table 1). Studies about the effect of medical humanities education on medical school students have also echoed the same findings [16–18]. For example, one study found that students perceived value in learning clinical communications skills after taking a medical drawing course [19]. Other students have reported that medical humanities projects provide important self-reflection time and joy outside of their typical coursework [20].

Some PDs were less confident in whether the medical humanities influenced performance in residency (‘No effect,’ ‘unlikely to improve their mastery of diagnosis and treatment’; Table 1). The study of medical humanities has long carried the burden of quantitatively proving its worthiness in medical training. This has proven to be a challenge given that the emotional impact of the humanities is difficult to capture with a numerical analysis [13]. However, the strong support (95%) of medical humanities research displayed in Question 1 of our survey indicates that it may not prevent a student from being seriously considered in both surgical and medicine residency programs (Figure 1a). Surprisingly, the sole respondent that answered ‘No’ to Question 1 was from psychiatry, a specialty that has historically attracted humanities-minded students [15].

Impressively, a majority of PDs (65%) said that doing medical humanities in addition to clinical research increased an applicant’s chance of being accepted (Figure 1a). This implies that programs may be increasingly seeking well-rounded applicants with diverse interests. This sentiment was further emphasized in qualitative responses. Responses included that the medical humanities ‘helps with … understanding of diversity,’ ‘broadens the view of the individual,’ and ‘may make the individual more interesting’ (Table 1). Not only may students stand out from their peers if they participate in humanities-based projects, but they could also have a broader, person-centered outlook on clinical care. Because of its inherent capability to promote self-reflection and critical thinking, the arts and humanities are considered an essential vehicle for delivering content related to diversity, equity, inclusion, and disability justice, which are topics increasingly emphasized in medical school curricula, admissions, and standardized testing [21–23].

PDs were split on whether medical humanities research should pertain to their specialty of interest or not, with most saying that they did not have a preference (Figure 1b). Rather than having projects that were in the relevant field, one psychiatry PD expressed that ‘[taking] a project from concept to publication’ was a more important marker of a good applicant (Table 2). Another psychiatry PD even emphasized that these projects did not need to take the form of a published article, saying ‘we would value the work of someone who published a personal essay as much as someone who did medical humanities research’ (Table 2). Different modalities of projects can be equally as valid as long as the student rigorously engages with the process.

Though the survey results demonstrated that medical humanities research may be a valid and potentially advantageous experience for an applicant, PDs were still hesitant to say that the medical humanities were an important selection criterion for their program overall (Figure 1b). However, 25% (1) of otolaryngology PDs, 17% (1) of ophthalmology PDs, and 40% (4) of psychiatry PDs agreed or strongly agreed that it was (Figure 1b). This is a high percentage of programs (25%) given that residency programs have only sparsely adopted the medical humanities into their actual curricula. It is possible that this number foreshadows a growing importance and prevalence of the medical humanities in residency programming. Applicants with medical humanities experience may be actively sought out for their skills in the coming years as programs seek to ramp up humanities integration into the clinical space.

## Limitations

Our study was limited by the small sample size (*n* = 20) and number of specialties. The response rate of 31% could potentially be a sign of selection bias among PDs. Future studies should pursue more specialties on a larger scale. It would also be beneficial to survey PDs beyond New York State. Programs in different states and settings (e.g., rural) may have different priorities for their applicants. Though our questions were designed to be as concise and clear as possible, there is always the possibility that participants misinterpreted or misread questions.

## Conclusions

Several studies have investigated the otolaryngology and ophthalmology residency application process from the perspective of logistical and financial barriers to students [24,25]. We are not aware of a previous study that has directly asked residency PDs what they are looking for in applicants. As a consequence, students are often left wondering what they need to do to be a good applicant. This results in students defaulting to what they perceive to be safe areas of research (i.e., clinical). This study was conducted in the hopes that students will read this and feel more assured in the decision to pursue medical humanities research on its own or in additional to clinical research. They should feel empowered in this choice whether they want to enter a competitive surgical field like otolaryngology and ophthalmology or a medical field like psychiatry. Doing so may even confer an advantage to individuals in the application process and in their residency and career performance.
